# Interactions of Dimethylarsinic Acid, Total Arsenic and Zinc Affecting Rice Crop Management and Human Health in Cambodia

**DOI:** 10.5696/2156-9614-10.26.200612

**Published:** 2020-05-28

**Authors:** Tom Murphy, Kim Irvine, Kongkea Phan, David Lean, Emmanuel Yumvihoze, Ken Wilson

**Affiliations:** 1 International University, Phnom Penh, Cambodia; 2 Thammasat University, Bangkok, Thailand; 3 Lean Environmental, Apsley, Ontario, Canada; 4 University of Ottawa, Ottawa, Ontario, Canada; 5 Texas State University, San Marcos, Texas, USA

**Keywords:** arsenic, zinc, rice, XRF, irrigation, drainage, fertilization, Cambodia

## Abstract

**Background.:**

In parts of Cambodia and in many other parts of the world, irrigation of rice with groundwater results in arsenic (As) accumulation in soil and rice, leading to health concerns associated with rice consumption. At times, some As is present as relatively nontoxic, non-regulated, dimethylarsinic acid (DMA). Low levels of zinc (Zn) have been found in rice from Bangladesh, Cambodia, and China where As levels in rice are high. Furthermore, there have been claims that Zn deficiency is responsible for stunting the growth of children in Cambodia and elsewhere, however in rural Asia, rice is the major source of Zn. Current data are inadequate for both Zn and DMA in Cambodian rice.

**Objectives.:**

The present study aimed to provide a preliminary evaluation of the relationship between the content of Zn and DMA in rice grain in Preak Russey, an area with elevated levels of As in groundwater and to improve the management of Zn deficiency in rice.

**Methods.:**

Rice agriculture was evaluated along the Mekong River in Cambodia. Analyses for metals, total As, and As species in rice and water were conducted by inductively coupled plasma mass spectrometry. Analysis of total Zn and As in soils and total Zn in rice were analyzed using X-ray fluorescence (XRF) spectrometry.

**Results.:**

Rice in Preak Russey had Zn concentrations less than a third the level recommended by the United Nations World Food Programme. There was a significant (p < 0.05) negative correlation between the Zn content of rice and DMA in rice with the lowest Zn and highest DMA levels occurring near irrigation wells, the source of As.

**Conclusions.:**

The highest levels of DMA in rice were associated with Zn deficiency in rice.

**Competing Interests.:**

The authors declare no competing financial interests

## Introduction

There are over 2.4 million people living in the arsenic (As)-contaminated zone of Cambodia.^1^ The worst sites have groundwater with more than 1000 μg/L of As or 100 times the recommended guideline suggested for drinking water according to the World Health Organization (WHO) and 10 times the guideline for irrigation water by the Food and Agriculture Organization of the United Nations (FAO).^2–4^ Decades ago, tens of thousands of wells were dug in Cambodia to reduce diarrhea, cholera, and other diseases that occurred due to drinking water from surface sources. Arsenic toxicity can be as subtle as impairment of intellectual development, but may also include congenital birth defects in children.^5^ In adults, arsenic toxicity may lead to amputation of limbs to remove cancerous growths and cancer mortality.^6^

In recent studies, all rice samples collected at 12 farms in Preak Russey exceeded the As content allowed for children in the European Union (EU) and seven of these farms had levels over twice this guideline.^7,8^ The United States Food and Drug Administration proposed a similar guideline for As in children's cereal.^9^ These guidelines and similar ones developed by the FAO/WHO specify or emphasize inorganic As as the regulatory parameter, not total As.^10^ In the rice rhizosphere, inorganic As is detoxified first to monomethylarsonic acid (MMA) and then dimethylarsinic acid (DMA).^11–13^ The American Agency for Toxic Substances and Disease Registry published data showing that DMA acid has 1/66th the minimal risk level toxicity of inorganic As.^14^ This is part of the reason that DMA is not regulated by Codex.^10^ There has been very little evaluation of how DMA influences or reflects the quality of rice as food. However, DMA is commonly associated with rice straighthead disease that reduces rice productivity and reflects disrupted plant physiology.^13,15,16^

**Abbreviations**
AsB: Arsenobetaine
CRMs: Certified reference materials
DMA: Dimethylarsinic acid
EU: European Union
FAO: Food and Agriculture Organization of the United Nations
ICP-MS: Inductively coupled plasma mass spectrometry
MMA: Monomethylarsonic acid
NIST: National Institutes of Science and Technology
XRF: X-ray fluorescence

**Figure 1:**
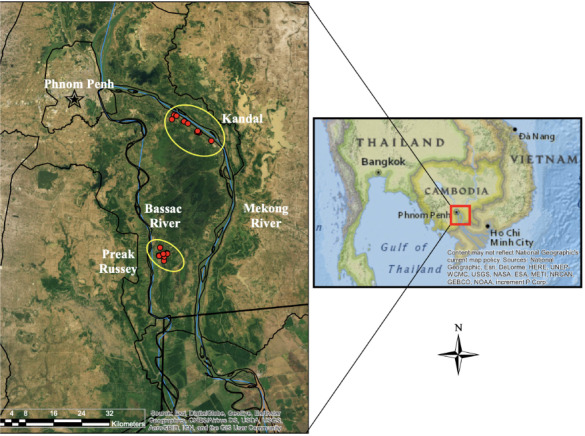
Map of the study area

The World Food Programme recommends that zinc (Zn) levels in rice should be higher than 60 μg/g for people eating 15–300 g/d of rice or more than 50 μg/g for people eating > 300 g/d of rice.^17^ Twenty-nine (29) brown rice samples from Preak Russey and Kandal *(Figure 1)*, and previously reported by Murphy *et al.,* had a mean of 17.1±4.9 μg/g for Zn, a third to a fifth of recommended values.^1^ Variance from levels recommended by the World Food Programme are common around the world *(Table 1).*

**Table 1:** Suggested Zinc Guidelines and Zinc Market Surveys of Rice

**Location**	**Lowest Zn, μg/g**	**Mean Zn, μg/g**	**Comments**
Guidelines			
Proposed 2017^17^	60	n.a.	World Food Programme
Codex 1987^18^	44.1	n.a.	Infants 0–6 months
Codex 1987^19^	14.7	n.a.	Infants 6–12 months
Proposed 2015^20^	17.6	n.a.	Infants 12–36 months
Country Surveys			
Bangladesh^21^	<5	39	Mean 4 districts, 23 fields
Bangladesh^22^	29	30	Control vs. + NPK Zn for 23 years
Cambodia	5.7	17.1 ±4.9	This study, 16 fields
China^23^	20.5	24.7	Control vs. + Zn - soil and foliar
India^24^	31.8	37	Zn Fertilization
India^25^	21.6	25.1	Control vs. + Zn 2 foliar
India^26^	12.7	17.5	N=38, XRF analysis
Iran^27^		28.6	N= 90, various rice, some enriched
Qatar^28^		12.5±5.35	N=56, white rice
Thailand^29^	17	23.8	Brown rice + foliar Zn
Uganda^30^		14	Mean of 4 districts

^17^ Proposed 2017 for all people consuming 150–300 g/d of rice

Abbreviations: n.a., not applicable; NPK, nitrogen, phosphorus and potassium; XRF, X-ray fluorescence.

Brown (or de-husked, but unpolished) rice typically has a higher concentration of Zn than white (or polished) rice, but the bioavailability of Zn in brown rice is lower due to phytate complexation.^31^ Phytates, other chelators in rice (nicotianamine, phytochelatin), and metal transport systems have very different controlling parameters than the geochemistry of groundwater. The correlations between metals observed in rice grain reflect both geochemical groundwater reactions and biochemical reactions in rice and its associated rhizosphere. Correlations are not proof of any reaction, but correlations coupled with appropriate sampling can lead to hypotheses to test for improved management of rice cultivation. In rural Cambodia, brown rice is often eaten because it stores better than white rice. Globally, there is considerably less information on Zn in brown rice than white rice. The present study is an extension of earlier analyses of Zn and As in Cambodian rice.^1^ The aim of the present study was to examine the importance of Zn to the detoxification of As by methylation in brown rice.

## Methods

**Figure 2:**
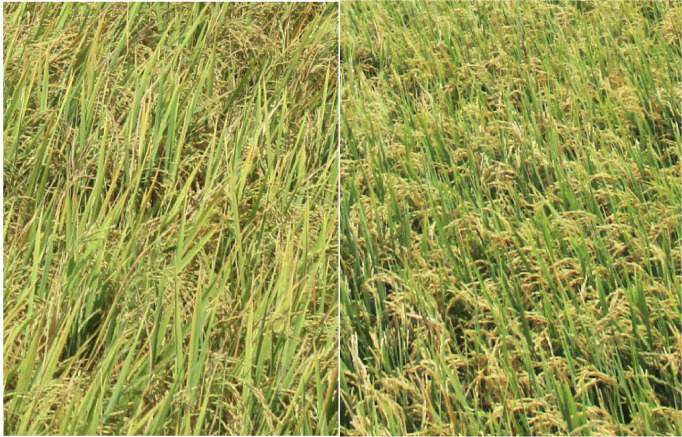
Chlorotic and healthy rice plants, 2016. Chlorotic Preak Russey-1 rice on the left and healthy Preak Russey-5 rice on the right

Figure 1 shows the study area. The As content of groundwater varies from very high in Preak Russey (mean of 953±349 μg/L total As) near the Bassac River to lower levels in the groundwater near the Mekong River (mean of 103±175 μg/L total As) in a control area, Kandal *(Tables 2 and 3).* Both areas are mainly flood plains with farms that have very similar agricultural techniques and as the Bassac River is a distributary of the Mekong River, the water source is similar. Monitored farms mainly use groundwater irrigation, but one farm in the present study (Preak Russey-15) had used only surface water for irrigation for 25 years. One farm in the present study had an effective system to remove >95% of As in groundwater prior to irrigation (Preak Russey-4).^1,32^ Our study primarily focused on farms using groundwater irrigation. Our data are not representative of the average farm in this area because most farmers use surface water and do not grow a second crop in the dry season using well water. Less than a third of the farmers in the present study grew a second crop with well water and all farmers grew one crop a year with rainwater. Most of the rice samples in the present analysis were collected from farmers in 2016 as part of an earlier International Development Research Center-funded project.^33,34^ Combines are used in this area for rice harvesting. The combines operate by concentrically moving from the outside of the field towards the middle, which effectively integrates the rice for sampling purposes. For all sites except Preak Russey-1 and Preak Russey-9, we collected three samples and used that as a composite for one analysis. Preak Russey-1, Preak Russey-5, Kandal-3 and Kandal-9 were composite means of 9 samples, each analyzed separately. Although there were small variations of the color of the rice in several fields, Preak Russey-1 farm was the only site with consistent and obvious chlorotic rice (*Figure 2*). The rice in Preak Russey-5 was chosen as a control to Preak Russey-1 because the rice looked greener, taller, and more prolific and both fields had a similar history of limited irrigation with groundwater (three years for Preak Russey-1 and one year for Preak Russey-5). The Preak Russey-1 and Preak Russey-5 fields were 100 m apart and grew the same rice variety (IR 85). The duration of groundwater irrigation varied from as little as one year to up to 20 years in Preak Russey and 34 years in Kandal. The soils most contaminated with As had been irrigated with groundwater for 9 and 13 years, respectively (Preak Russey-2 and Preak Russey-9).^34^ All fields had similar alluvial clay soils.^8,33^ Two farms in the control area, Kandal-3 and Kandal-9, also had 9 replicate samples, to balance the comparison of the control area to Preak Russey. Occasionally, the full matrix of analyses is missing data, mainly because some farmers feared we would interfere with their use of groundwater for irrigation and became uncooperative.

**Table 2:** Groundwater Well Arsenic and Location—Kandal

**Farm**	**AsB**	**As (III)**	**DMA**	**As(V)**	**Total As**	**Easting**	**Northing**
Kd-1	nd	142	nd	307	448	105° 13′22.08″	11° 24′ 38.88″
Kd-1b	nd	327	nd	276	603	105° 13′30.08″	11° 24′ 19.28″
K.d-2	nd	40	nd	145	185	105°10 36.78″	11° 25′ 42.49″
Kd-2b	nd	0.1	nd	47	47	105°11′ 31.62″	11°25′ 56.07″
K.d-3	nd	1.1	nd	71	72	105° 11′ 20.0″	11° 26′58.80″
K.d-3	nd	0.1	0.1	0.5	I	105° 11′ 20.11″	11°. 26′ 00.43″
Kd-4	nd	nd	nd	7.5	8	105° 09′ 09.4″	11° 27′ 39.8″
Kd-5	nd	nd	9	9	0	105° 09′ 05.3″	11° 27′ 36.8″
K.d-6	nd	0.3	1.1	19.5	21	105° 09′ 45.8″	11° 27′ 19.4″
K.d-7	nd	nd	nd	11	10	105° 07′ 46.92″	11° 28′42.65″
K.d-8	nd	2.4	8.5	69	72	105° 07′ 38.2″	11° 28′ 38.2″
K.d-9	nd	nd	nd	0.4	0	105° 07′ 17.3″	11° 27′ 58.6″
Kd-9b	nd	nd	nd	0.1	0	105° 07′ 30.2″	11° 28′ 11.5″
Kd-9c	nd	0.0	0.0	0.5	1	105° 07′ 30.2″	11° 28′11.5″
Kd-10	0.14	79	nd	26	95	105° 11′ 21.98″	11° 26′01.63″
Kd-10b	nd	43	nd	29	71	105° 11′ 21.8″	11° 26′ 03.3″
Mean		58		64	103		
SD		100		97	175		

Concentrations are means in (μg/kg.

Abbreviations: Kd, Kandal well; nd, not detected.

**Table 3:** Groundwater Well Arsenic and Location—Preak Russey

**Farm**	**AsB**	**As (III)**	**DMA**	**As(V)**	**Total As**	**Easting**	**Northing**
PR-1 adjacent	nd	1163	nd	298	1461	105° 06′ 5.36″	11° 07′ 3.10″
PR-1b	nd	57	nd	108	165	105° 06′01.96″	11° 06′ 59.17″
PR-2	nd	625	nd	411	1037	105° 06′ 07.016″	11° 06′ 56.109″
PR-2b	nd	765	nd	981	1446	105° 06′ 03.62″	11° 07′ 30.83″
PR-2 chy*	nd	655	nd	218	873	105° 06′ 07.016″	11° 06′ 56.109″
PR-4	nd	66	nd	567	633	105° 05′25.97″	11°06′56.80″
PR-4 adjacent	nd	43	nd	707	750	105° 05′25.97″	11° 06′56.80″
PR-6	nd	990	nd	171	1161	105° 06′ 04.183″	11° 06′ 57.624″
PR-7	nd	603	nd	176	779	105° 06′ 04.92	11° 07′ 06.60″
PR-7b	nd	396	nd	312	709	105° 05′ 47.8″	11°07′ 52.8″
PR-8	nd	265	nd	273	538	105°.05′28.3″	11°08 10.2″
PR-9a	nd	995	nd	276	1270	105° 06′ 03.671″	11° 06′ 48.605″
PR-9b	nd	360	nd	475	834	105° 06′01.294″	11° 06′ 48.879″
PR-9c	nd	795	nd	290	1085	105° 06′ 06.869″	11° 06′ 47.280″
PR-10	nd	582	nd	463	1044	105° 05′ 17.01″	11°06′ 56.63″
PR-10b	nd	475	nd	476	951	105° 05′ 19.719″	11° 06′ 53.127″
PR-12	nd	921	nd	327	1248	105° 06′31.514″	11° 07′ 19.94″
PR-13	nd	583	nd	305	887	105° 06′ 33.206″	11° 07′ 15.488″
PR-14	nd	519	nd	451	970	105° 06′ 34.822″	11° 07′ 13.864
PR-16	nd	757	nd	464	1221	105° 05′58.136″	11° 07′ 11.919″
PR-19	0.01	1504	nd	202	1706	105° 06′21.26″	11° 07′ 12.29″
Mean	NA	617	NA	336	953		
PR							
SDPR	NA	377	NA	154	349		

Concentrations were means in μg/kg.

Abbreviations: PR, Preak Russey; NA, not available; nd, not detected; SDPR, standard deviation of the drainage ditches in Preak Russey, Mean PR, mean of 5 samples of water draining from Preak Russey rice fields.

*Chy is the herbal plant growing at this site.

The present paper only evaluated brown rice. Rice was air dried and dehusked by hand. For As speciation analysis rice was ground with a mortar and pestle until the powder could pass a 100-μm mesh. For the X-ray fluorescence (XRF) analysis, rice was ground with a generic food processor (Electrical Powder Grinder DE-200 g). Tools were cleaned between samples, first with a wet cloth and then with a dry cloth. To avoid overheating, rice was ground for less than 30 seconds. For samples larger than 100 ml, this processor worked well, but smaller samples required long delays to avoid heating the sample. The best food processors are expensive and for developing countries, a mortar and pestle is adequate. Further details can be found in earlier publications.^8,33^

We used two different Niton XL3t GOLDD handheld XRF analyzers and a Bruker S1-600 Titan XRF analyzer for analysis. Different XRF units were used in the present study due to availability issues.^1^ We used a two-minute analysis time on soil mode. All samples were processed using the sample cup method recommended by Thermo Fisher Scientific with Mylar film (Figure 2 in Murphy *et al*.).^33^ For rice, the following certified reference materials (CRMs) were used: 180–600 (soil), National Institutes of Science and Technology (NIST) 1568b (rice flour), CRM NIST 2710 (soil), and silica for a blank for quality assurance/quality control purposes.^33,35^ The measured mean and standard deviation XRF analysis of CRMs were within 9±12% of the certified values *(Table 4).*^1^ For XRF analysis of rice, the coefficient of variation was 6.87±7.47% for 44 samples. The CRM (NIST 1568b) is reported to have 19.42±0.26 ppm of Zn and was measured as 18.7±1.5 ppm in the present analysis.

**Table 4:** Certified Reference Material Analysis for Zinc^1^

**Sample**	**Mean certified**	**Mean measured**
NIST 1568b (rice)	19.42±0.26	19.7±0.8
180–600 (soil)	46±3	39.7±1.5
NIST 2710 (soil)	4180±15	4203±33

All values are means of three analyses ± standard deviation.

All values are presented as μg/g.

### Analysis

All water and rice samples were shipped to Canada for analysis. Trace elements in the water samples including As were measured by inductively coupled plasma mass spectrometry (ICP-MS). Arsenic speciation of rice was conducted at the University of Ottawa using an Agilent 1200 Infinity LC system connected to an Agilent 7700x ICP-MS according to the United States Environmental Protection Agency Method 200.8.^36^ Arsenic species were quantified using the method developed by Agilent Technologies, including arsenic(III), arsenic(V), MMA and DMA.^37^

For analysis of As speciation, rice samples were composited and portions of the composite were mixed with a 0.28 M nitric acid solution and heated at 95°C for 90 minutes. The extracts were initially diluted with deionized water, centrifuged, filtered, and then diluted further while adjusting pH prior to analysis by high pressure liquid chromatography-ICP-MS.^38^

### Reagents and standards

All reagents were analytical grade. Arsenic standards and other reagents were purchased from Sigma-Aldrich and Spex CertiPrep. Stock solution of 1000 mg/l of arsenite (Spex CertiPrep, Cat#SPEC AS3M), and arsenate (Spex CertiPrep, Cat#SPEC-AS5M), and 10 mg/L of DMA (Spex CertiPrep, Cat# SPEC-AS-DMA) and MMA (Spex CertiPrep, Cat# SPEC-AS-MMA) with a certified value of As value traceable to NIST Standard Reference materials were purchased from Spex CertiPrep. Arsenobetaine stock solution of 1000 mg/l was prepared by weighing and dissolving arsenobetaine salt. Ten (10) mM of ammonium phosphate dibasic was prepared by dissolving ammonium phosphate dibasic (Sigma, Cat#379980-100G) in Milli-Q water (Millipore) and pH adjusted to 8.25 with 28% ammonium hydroxide solution (Sigma, Cat#: 338818-100ML). Mobile phase was filtered through a 0.45 micron-filter before use.

### Instruments

The chromatographic separation of arsenite (As(III)), arsenate (As(V)), MMA, DMA and arsenobetaine (AsB) was performed using a 10 mM ammonium phosphate dibasic buffer with pH adjusted to 8.25 on an Agilent 1200 Infinity LC system consisting of a 1260 Isocratic pump and 1260 Auto sampler. The LC system was linked to the Agilent 7700x ICP-MS via Peek tubing and equipped with a low flow Micro Mist Nebulizer and quartz, low-volume Scott-type double-pass spray chamber. The mobile phase was pumped at 1 ml/min and injection volume was set at 100 μL.

Instrument run conditions are listed in Table 5.

**Table 5:** Instrument Run Conditions

Column	PRP-X100, 10 um × 250 mm × 4.6 mm id
Mobile phase	10 mM ammonium phosphate dibasic, pH= 8.25
Flow rate	1 ml/min
Injection volume ICP-MS conditions	100ul
Radio frequency power	1550 [W]
Plasma gas flow	15.0 L/min
Sampling depth	8.0 mm
Nebulizer gas flow	1.1 [L/min]
Spray chamber temperature	2 [C]
Collision cell gas	Helium, 2.0 ml/min
Data acquisition mode and isotope monitored	Time-resolved analysis: m/z for 75 As^+^ and m/z 77 for 40Ar^+^37Cl^+^
Integration time per isotope	0.8 s(m/z 75), 0.2 s(m/z 77)

Statistical analyses of all data were done using Excel and VassarStats.^39^ The measured As species in the CRM for ICP-MS analysis were always within the standard deviation of the CRM *(Table 6).* The coefficient of variation of replicate As species analysis for individual rice samples was usually less than 10% *(Table 7).* The coefficient of variation was highest for As (III).

**Table 6:** Quality Assurance Quality Control for Rice CRMs and Lab Methods

	**AsB**	**As(III)**	**DMA**	**MMA**	**As(V)**	**Total Inorganic As**
Blank	0.1 ±0.01	0.00	0.00	0.00	0.17±0.01	NA
CRM standard	NA	NA	180±12	11.6±3.5	NA	92±10
CRM lab	3.5±0.1	41.5±8.5	182±5.7	12.1±0.7	53.8±8.4	95.4±0.1
Check standard	NA	5	5	5	5	5
Check standard lab	NA	4.48±0.11	5.29±0.11	4.78±0.08	4.91 ±0.2	NA

Concentrations were means in μg/kg.

Abbreviations: NA, not available.

**Table 7:** Quality Assurance Quality Control for Replicate Arsenic Species Analysis of Individual Rice Samples

	**AsB**	**As(III)**	**DMA**	**MMA**	**As(V)**
PR-1	4.4±6.8	31.8±10.4	71.2±4.3	5.5±0.4	216.3±6.8
PR-12	2.5±0.3	28.1±. 1	346±1.5	4.5±0.2	175.4±14.2
PR-13	5.1±0.3	25.1 ±22.2	305.7±9.9	4.6±0.2	256.9±53.7
PR-15	2.4±0.6	8.9±2.2	17.0±0.7	1.4±0.0	117±0.6
PR-18 NW	1.3±0.1	30.7±1.4	204.3±0.6	4.7±0.3	216±8
PR-18 FW	1.0±0.1	22.7±6.4	12.7±4.1	3.3±0.1	180±10.2
CV	7.76	28.24	2.56	4.05	6.08
CVSD	7.93	28.86	2.17	2.48	6.55

Concentrations were means in μg/kg.

Abbreviations: PR, Preak Russey; NW, near irrigation well; FW, far from irrigation well; CV, mean of all coefficients of variation; CVSD, standard deviation of the means of all coefficients of variation.

## Results

The most important difference between the Preak Russey farms and the Kandal control farms was the mean concentration of inorganic As in 21 wells in Preak Russey (953±349 μg/L) relative to 16 wells in Kandal (103 ± 175 μg/L) *(Tables 2 and 3).* This difference was highly significant (p < 0.0001, Mann-Whitney U test). Only two wells in the 10 Kandal farms showed levels greater than 100 μg/L of total inorganic As; whereas none of the wells in Preak Russey had less than 400 μg/L of total inorganic As. Of the three measured forms of organic As (AsB, DMA and MMA), only six of the 37 well water samples from both Preak Russey and Kandal had detectable organic As and only two had more than 2 μg/L of organic As (DMA, 8.5 μg/L in Kandal-11 and 9 μg/L in Kandal-5). Total arsenic, As(V) and As(III) concentrations were all significantly higher in Preak Russey than in Kandal (p<0.0007). Monomethylarsonic acid was only detected in one well at 0.1 μg/L.

Conversely, in 5 water samples from ditches receiving drainage seeping via the soils from rice paddies in Preak Russey, DMA, MMA and AsB were readily measured *(Table 8).* In these drainage ditch samples, DMA and MMA were 2.8% and 2.0% of the total As in solution (total As mean 53.04±33 μg/L), respectively. The detection limit for DMA was less than 0.1 μg/L and the mean concentration of DMA in the drainage ditches was more than 60 times higher than in the well water samples. This indicates that DMA forms in soils, likely the rice rhizosphere, and does not come directly from groundwater.^11–13^

**Table 8:** Arsenic Speciation of Drainage Ditches

	**AsB**	**As(III)**	**DMA**	**MMA**	**As(V)**	**Total As**
PR-2 ditch	1.53	3.07	8.38	1.08	56.9	70.9
PR-4 ditch	0.17	1.27	0.47	0.93	2.91	4.92
PR-10 ditch a	1.04	15.36	8.57	0.093	22.92	48.82
PR-10 ditch b	0.74	6.23	7.11	1.31	30.45	45.85
PR-10 ditch c	0.58	78.03	6.18	1.02	29.31	94.67
Mean	0.81	20.79	6.14	28.49	0.89	53.04
SD	0.51	32.46	3.32	19.33	0.46	33.31

Concentrations are μg/L.

Abbreviation: PR, Preak Russey.

The metals detected in irrigation wells of Preak Russey and Kandal control farms were quite different. The concentrations of As, barium (Ba), iron (Fe), strontium (Sr) were significantly higher in Preak Russey (p = <0.0001, 0.0005, 0.0013, 0.0003, respectively) than in the Kandal control farm wells *(Table 9).* Conversely, the concentration of lead (Pb) and Zn were significantly higher in the Kandal farm wells (p = 0.002, 0.0129, respectively) than in the Preak Russey wells *(Table 9).* The mean content of Zn in groundwater irrigation water of Preak Russey farms was 4.0±4.0 μg/L. In Preak Russey, the average volume of irrigation water was 11 600 cubic meters per hectare. The calculated loading of Zn from irrigation is about 1% of the Zn fertilization rate suggested by the Rice Institute of 10–25 kg Zn sulfate water per ha.^40^ The wells in Kandal had an average concentration of 9.4 μg/L of Zn, which although significantly higher than Preak Russey, is still less than 3% of the recommended fertilization rate. This influx of Zn from irrigation with groundwater to rice was inadequate to sustain rice growth. At times the groundwater smelled like sulfides, which would precipitate Zn in the aquifer. Similarly, the XRF analysis of eleven inorganic fertilizers and interviews with farmers indicate that the loading of Zn in Preak Russey was 0.15 kg/ha as hydrated Zn sulphate, which is less than 2% of the recommended fertilization rate of the Rice Institute.^1,40^

**Table 9:** Metal Content of Irrigation Wells

**Metal**	**Al**	**Mn**	**Fe**	**Zn**	**As**	**Sr**	**Ba**	**Pb**	**Mg**
Kandal wells (n=16)									

Mean	136	721	3711	9.4	136	299	252	6	19387
SD	170	682	3667	12.0	236	98	194	21	6678

Preak Russey wells (n=21)									

Mean	25	514	9483	4.0	1097	574	1057	0.09	25649
SD	20	300	6972	4.0	394	304	731	0.09	13714

Probability of Kandal samples not being different than Preak Russey samples									

p value	0.0582	0.4483	0.001	0.0129	0.0001	0.0003	0.0005	0.0002	0.129:

Concentrations presented in μg/L.

### Arsenic and zinc concentrations in rice

Differences in the As content of rice in farms of Preak Russey and Kandal were substantial but not statistically significant. The mean concentration of inorganic As in brown rice in 12 farms in Preak Russey was 203±51 μg/kg, and 144±34 μg/kg in 10 farms from the Kandal sites *(Table 10).* The contents of total As, inorganic As, MMA and DMA were higher in the farms in Preak Russey than the Kandal control site *(Table 10).* Conversely, the concentrations of As(III) and AsB were a little higher in the control sites of Kandal *(Table 10).* The percentage of inorganic As was higher in the Kandal control site whereas the percentage of DMA of total As was higher in Preak Russey *(Table 10).*

**Table 10:** Summary of Arsenic Speciation of Brown Rice

	**AsB**	**As(III)**	**DMA**	**MMA**	**As(V)**	**Total of all As species**	**Total inorganic As**
All farms combined							

Concentration	5	30	84	9.2	146	265	177
SD concentration	4.7	16	92	14	50	134	52
Range	0.93–28	8.5–68	10–34d	1.1–19	52–257	106–597	41–304
% of total As	2.2	13	25	3.8	60	NA	75
SD %	1.7	16	15	5.7	13	NA	19

Kandal control farms							

Concentration	5.4	34	40	2.7	111	193	144
SD concentration	5.9	16	31	1.1	29	67	34
Range	1.1–22	8.5–53	10–108	1.1–4.8	52–160	106–322	41–197
% of total As	2.6	18	20	1.4	59	NA	74
SD %	1.7	9	9	.4	10	NA	19

Preak Russey farms							

Concentration	4.7	28	124	15	174	326	203
SD concentration	3.6	17	111	17	45	148	51
Range	0.6–28	8.8–68	13–346	1.0–19	117–257	145–597	89–304
% of total As	2.0	9	29	5.8	59	NA	62
SD %	2.2	4.9	17	7.3	10	NA	16

Probability of no difference in arsenic species between Kandal and Preak Russey							

p-value	0.0643	0.0735	0.0643	0.0735	0.0735	0.0735	0.1685

Comparison to Chinese brown rice^41^							
	NA	NA	NA	NA	NA	255	209

Concentrations presented as means in μg/kg.

Rice was processed from 12 farms in Preak Russey and 10 in Kandal sites.

Abbreviation: NA, not applicable or not available.

p-value = Mann-Whitney U test.

**Figure 3:**
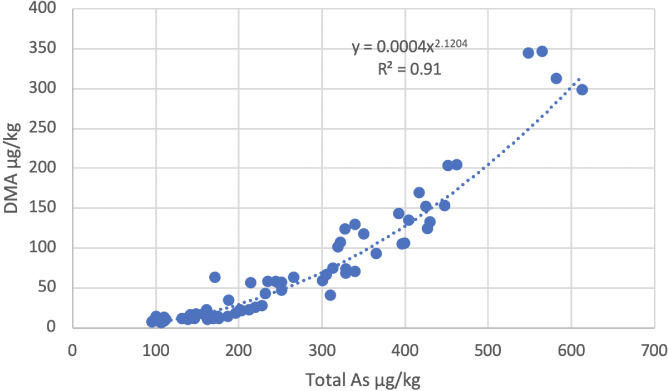
Effect of total arsenic on DMA in rice; full data set of all 70 samples that were processed for arsenic speciation analysis

The statistical comparison of rice from farms in Preak Russey and Kandal was limited by the number of farms that could be processed. Additional insight can be found by combining data. In Figure 3, the 70 samples of all rice processed (including replicates and quality control analyses) for As speciation from Preak Russey and Kandal illustrate the strong relationship between the DMA content of rice and total As content (R^2^ = 0.91). Moreover, the correlation appears to follow a power function (Y= 0.0004x^2.1204^) so that the samples with highest total As have much more DMA then would be expected by a linear relationship. The relationship improves slightly to R^2^ = 0.95 (data not shown) by only plotting the 43 samples (including replicates and quality control analyses) from Preak Russey. In all rice samples, the dominant form of measured organic As was DMA (25±15% of total As). The DMA content of Preak Russey (124±111 μg/kg) was higher than for Kandal (40±31 μg/kg), but the difference in concentration was only significant at p = 0.064 (Mann-Whitney U test).

**Figure 4:**
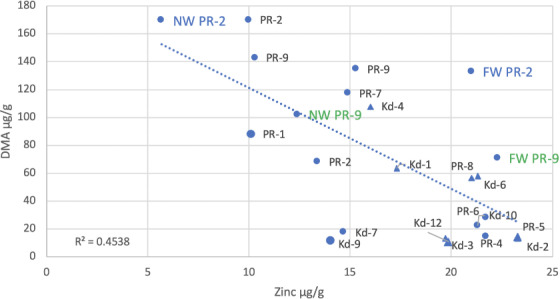
Dimethylarsinic acid content of rice versus total zinc content of rice Data from both Kandal (Kd) and Preak Russey (PR) sites. Abbreviations: FW, far from irrigation well; NW, near irrigation well. Near irrigation well Preak Russey-2, far from irrigation well Preak Russey-2, near irrigation well Preak Russey-9 and far from irrigation well Preak Russey-9 and single samples either very close to the irrigation well or as far as possible from the well in the same field. Other samples are integrated samples collected by the combine (see methods). Note that the pairs of wells in Preak Russey-2 and Preak Russey-9 in Figure 4 have larger fonts with different colors. This reflects the importance of the wells as the source of arsenic and importance of sampling proportional to the wells. indicates farms with cows.

When all of the data were analyzed, there was a significant negative correlation between the Zn content of rice grains and the DMA content of the grains (p < 0.05, R^2^ = 0.45) *(Figure 4).* The correlation between total inorganic As and Zn in the rice was not significant (p > 0.08, R^2^ = 0.31, not shown). The correlation between As(V) and Zn in the rice was not significant (p > 0.1, R^2^ = 0.22, not shown). The correlation between As(III) and Zn was also not significant (p > 0.1, R^2^ = 0.10, essentially random and not shown). Since we were monitoring independent farms, it was not always possible to collect samples from farms in the same fashion, but there were always at least three subsamples aggregated per sample. Four of these samples shown in Figure 4 were means of analyses of 9 samples (Preak Russey-1, Preak Russey-5, Kandal-3, Kandal-9) represented with slightly larger symbols. If these four samples were removed from the data set to resolve potential concerns about variance affecting statistical analysis, the remaining data set would still result in a significant correlation (p < 0.025, R^2^ = 0.57). In fact, the correlation is slightly higher without these four sites, which reflects the removal of the outlier sample Kandal-9. Two of the biggest outliers in Figure 4, Kandal-7 and Kandal-9, from the Kandal area did not have cows. The other seven farms from Kandal had cows. Kandal-9 was a mean of 9 samples so the outlying characteristic for this site was strongly developed.

The variability in assessment of DMA versus Zn can be reduced by focusing on the farms in the curvilinear portion of *Figure 3*, i.e. more than 500 μg/L of As, the Preak Russey area. As a result, the R^2^ value increases to 0.52 (p < 0.05, not shown). These are the farms reflecting increased concentrations of DMA in rice, associated with high levels of As in irrigation water. Since DMA can reflect rice straighthead disease, it seems appropriate to evaluate the rice potentially reflecting this physiological disorder.^16^

**Figure 5:**
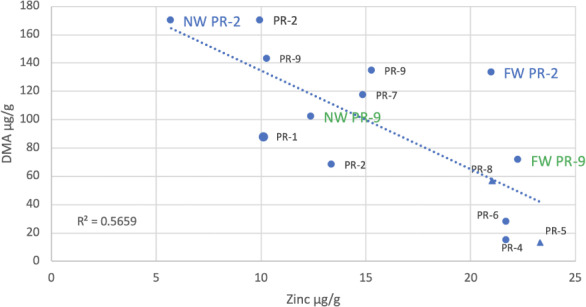
Total zinc content of rice versus DMA content of rice from Preak Russey (PR) sites Abbreviations: FW, far from irrigation well; NW, near irrigation well. Near irrigation well Preak Russey-2, far from irrigation well Preak Russey-2, near irrigation well Preak Russey-9 and far from irrigation well Preak Russey-9 and single samples either very close to the irrigation well or as far as possible from the well in the same field. Other samples are integrated samples collected by the combine (see methods). Note that the pairs of wells in Preak Russey-2 and Preak Russey-9 in Figure 5 have larger fonts with different colors. This reflects the importance of the wells as the source of arsenic and importance of sampling proportional to the wells. indicates farms with cows.

The relationship with DMA and Zn is significant. The responses would be stronger if other variables could be controlled. The main cause of variability is the collection of samples relative to the irrigation wells. Rice collected nearest the wells had the least Zn and the highest total As (Preak Russey-2 and Preak Russey-9) *(Figures 4 and 5).* The second most important cause of variability was the presence of cows at the sampled farms. The presence of cows increased the Zn in rice by 50% to 100%. There were significant differences in Zn levels in rice according to whether or not the farmers raised cows (Mann-Whitney U test, p <0.05) *(Figure 4).* The only sample with a level of Zn greater than 20 μg/g that did not have cows or was located more than 90 m from the well was sample Preak Russey-4, which had a treatment ditch that removed >95% of total As prior to irrigation.^8^

Preak Russey-1 was the only farm with obvious chlorosis of rice and warranted enhanced sampling. Preak Russey-5 was picked as a control to Preak Russey-1. Chlorotic rice can be seen in Figure 2.^1^ The DMA content of the rice from Preak Russey-1 was significantly higher than its control field of Preak Russey-5 (t-test, p <0.0001), but was lower than the DMA content of rice in Preak Russey-2 and Preak Russey-9 *(Figure 4).* The mean Zn content of rice from Preak Russey-1 was quite low (10 μg/kg). Rice from Preak Russey-5 had 23 μg/kg of Zn, but two other farms (Preak Russey-2 and Preak Russey-9) had levels of Zn similar to that of Preak Russey-1 *(Figure 4).* Moreover, Zn deficiency in rice with respect to a desirable content for human consumption occurred in healthier looking rice, i.e. Preak Russey-2 and Preak Russey-9. The reported rice productivity in this study was the highest in Preak Russey-9 (8 tons per ha) and Preak Russey-2's reported productivity (6–7 tons per ha) was within the top third of the reporting farms. Our field observations confirm these two farms were productive. Conversely, the reported productivity of Preak Russey-1 from 2015 was only 10% less than Preak Russey-2 and Preak Russey-9, but the crop in 2016 looked much less productive (*Figure 2*).^1^

The As(III) content of the rice in Preak Russey-1 was significantly higher than all other rice samples (p < 0.005) *(Table 11);* moreover, it was four times higher than the sample with the 2^nd^ highest content of As(III). This higher concentration of As(III) likely reflects a lower redox in the soils of Preak Russey-1 which may reflect a greater degree of stagnation or longer time of stagnation. The only obvious observation of relevance to this difference between Preak Russey-2, Preak Russey-9 and Preak Russey-1 is that the drainage ditches near Preak Russey-2 and Preak Russey-9 were wet more than twice as often as the ditch at Preak Russey-1.

**Table 11:** Arsenite Content of Chlorotic Preak Russey-1 Rice Relative to Other Rice in this Study

**Site**	**Mean As(III)**	**SD**	**Number of samples**	**% Total As**
PR-1	80.3	23	9	30.7
PR-2	21.5	12	4	5.4
PR-5	14.4	18.2	9	9.1
PR-9	18.7	5.7	5	5.6
All samples	13	16	70	4.9

All As values in μg/kg.

## Discussion

All samples analyzed in the present study complied with the 2014 Codex guidelines of 350 μg/kg of inorganic As for brown rice *(Table 10).*^42^ It is interesting that the average total inorganic As of 446 brown rice samples in China was 209 μg/kg, essentially the same as the average in Preak Russey, 203 μg/kg.^41^ Moreover, the authors of this Chinese review of As in rice stated that the Codex guideline provides less protection from As assimilation from rice than is recommended by the WHO for As in drinking water (10 μg/L). They proposed that additional restrictions on As in food are required.^3,41^ We believe that Preak Russey rice irrigated with groundwater has the highest concentration of As in rice reported in Cambodia.

In 2016, the EU imposed a much stronger guideline for inorganic As in children's food of 100 μg/kg.^7^ The same guideline is proposed for American children's food.^9^ One senior scientist has proposed that for children, the guideline for inorganic As in food should only be 50 μg/kg.^43^ The average for rice in 12 farms in Preak Russey had double this new EU guideline of 100 μg/kg inorganic As and some farms had three times this guideline *(Table 10).* Based on our household surveys, more than 90% of the farmers in Preak Russey understood that As is toxic and more than half of them kept rice for themselves that was grown only with surface water that is low in As. However, some families ate rice grown with groundwater irrigation. Furthermore, 6 out of 10 farms in the control area (Kandal) with a mean of 101±182 μg/L of total As in groundwater wells had rice that failed this new EU guideline.^7,8^

The rationale for this new EU guideline is based on the fact that children are much more susceptible to As and other toxins.^43,44^ Several children in Preak Russey suffer from congenital birth defects, but there has not been a recent assessment of their health (personal observation). In 2016, a piped water supply was made available in Preak Russey for drinking water. Many, but not all farmers believe that the piped water solved all their As problems. The source of the piped water supply is the Tonle Bassac River. It is treated with sand filters.^45^ In terms of As management, the piped water supply to Preak Russey was a very effective step forward. With our data of the two main sources of As (water and rice) it can be estimated that the piped water reduced the farmer's exposure to As by 95%; resulting in much lower levels of arsenic toxicity.^1^ Other aspects of the drinking water supply in this region are less clear and monitoring should reflect the ongoing development of Phnom Penh. In addition, periodic reduced water flows of the Tonle Bassac River decrease the dilution of pollutants. One limitation of sand filters that might become important is that they can convert ammonia to nitrate which is toxic to human infants.^46^ Sewage treatment in Phnom Penh has mostly relied on disposal into wetlands but such wetlands are rapidly being infilled with river sand to produce urban property.^47^ Management of As is still incomplete. Farmers are not able to assess some of the more subtle aspects of As toxicity such as cancer, impaired of intellectual development of children, other impaired neurological functions or a weakened immune system,^5,48,49^ nor can professionals distinguish toxicity reflecting historical exposures to As from ongoing low doses of As. Induction of cancer from As can take 10–30 years.^49^ It is very likely that cancers induced by As will still occur in Preak Russey.

The worst of the As toxicity has been abated in Preak Russey, and rice qualifies easily for export with Codex guidelines. However, newly developed EU guidelines for As in rice should be considered in Cambodia to better protect children. This health debate is complex but our household interviews indicated the farmers understood very well the need to have suitable water for irrigation.

Rice straighthead disease warrants more attention. In the only farm with severe chlorosis, Preak Russey-1, the As(III) content of rice grains was much higher than 3 other farms with 9 replicates (Preak Russey-5, Kandal-3, and Kandal-9, p >0.005).^1^ The higher As(III) content in the rice likely indicates lower redox of soils. To clarify the pathology, it would be important to measure the redox of the soil, rice productivity, proportion of rice with empty grains, Zn content of the rice and As speciation of the rice. There were also small areas in the field with less extreme yellowing of rice and some empty rice grains that could be potentially linked to the observed variation of As(III) content in the rice grain.^1^ Such sampling could be guided by visual inspection of rice and redox analysis of the soil done immediately in the field.

### Zinc in rice

Compared to As toxicity, Zn deficiency is not well understood by the farmers in Preak Russey. Zinc deficiency might have contributed to the chlorosis of Preak Russey-1 rice, but chlorosis appears more complex than just the concentration of Zn. The guidelines for Zn in rice are less well known than those for As in rice or irrigation water. The Codex guidelines for Zn in children's food are outdated and not commonly cited.^18,19^ The Codex regulations for Zn passed in 1987 were designed for commercial infant formula. However, it is relevant that the rice in Preak Russey does not pass these Codex guidelines and rice is the major component of children's food. Surveys in 2016 of children's food produced in the EU indicated that the new EU guidelines for As were being effectively implemented. If the World Food Programme Zn advisory were fully implemented, there would likely be many public reviews of the new Zn guideline. The proposed guidelines for Zn in rice by the World Food Programme are 2–3 times higher than the average Zn content commonly found in rice around the world, including Cambodia *(Table 1).* Previous studies have stressed that Zn deficiency is a major concern in Cambodia and is responsible for stunting the growth of about 40% of Cambodian children.^50–53^ There are several processes that impair Zn bioavailability and a full review is beyond the scope of this paper. Arsenic is associated with Zn deficiency in Bangladesh, Cambodia and China.^1,21,54^ In our previous study, Zn deficiency in rice occurred in Preak Russey soils containing 85±9 μg/g of Zn (n=75) and Kandal soils containing 78±12 μg/g of Zn (n=26).^1^ The suggested critical Zn deficiency threshold is ~10 μg/g in soils and the suggested baseline for good Zn nutrition of soils is 60 μg/g.^21^ We lacked the resources to measure productivity directly, but it does not appear that Zn was a major regulator of rice productivity. This is important and needs further resolution. If the farmers were to fertilize with Zn and it did not increase productivity, they would need an incentive to fertilize to enhance the Zn content of rice. Post-harvest augmentation of rice with Zn is beyond the scope of the present study but should be considered as well. The present study indicates that the Zn deficiency in rice in Preak Russey seems to reflect irrigation with groundwater. The probability of the effect of As toxicity and Zn deficiency having an additive negative effect on human health is high.

The apparent impairment of Zn bioaccumulation by As should be evaluated in more geographic areas. In Cambodia, much of the required clarity for management could be obtained with simple XRF analysis of the rice grain and associated analysis of As in irrigation wells by analytical kits or atomic absorption spectrometry. Globally, the DMA content of rice has a distinct geographic distribution whereby some areas produce rice with mainly inorganic As, but other sites produce rice with significant concentrations of DMA.^13,55^ The deficiency of Zn in rice also has distinct geographic distributions. However, in part because developing countries lack the laboratories for As speciation, there is little if any data on both DMA and Zn in rice as reported here and therefore geographic trends cannot currently be established. The microbial reactions in soils change as stagnation by flooding consumes oxygen; the biochemical reactions are closely linked by geochemistry. Sulphate-reducing bacteria and methanogens mediate As methylation and demethylation in paddy soils.^56^ Microbial sulphate reduction produces sulphide which precipitates Zn. Methylation produces DMA and some volatilization of As via an extra methylation step. Variations in the degree of reduction and microbes are expected, but the geochemistry is strongly linked and rice with DMA can be expected to be Zn deficient. There will be other reasons for the widespread occurrence of Zn deficiency in Cambodians and populations in other countries. Inadequate irrigation, poor field drainage, depleted soil organic content and other suboptimal farm management practices should be investigated to alleviate Zn deficiency.

We need to stress that Zn deficiency in areas contaminated with As likely enhances As toxicity. Zinc is an essential component of more than 300 enzymes and some, like superoxide dismutase, are essential in detoxification of As.^55^ More than 10 years ago, Sampson *et al*. reported that for unknown reasons, As toxicity developed faster in Cambodia than other countries.^2^ Zinc deficiency may be one factor supporting Sampson's hypothesis. Zinc is also essential in the immune system and thus Zn is important in resistance to disease.^48,57^ An Italian study on the enhanced effect of air pollutants on the Covid-19 virus is very relevant to Preak Russey.^58^ The exposure of Cambodian farmers to soils/dust near their irrigation wells with as much as 95 mg/kg of As could weaken their resistance to infections, including the Covid-19 virus.^32,58^ In the Netherlands, property owners with soils containing more than 55 mg/kg of As must begin management with a professional analysis of the site to consider remediation and reduction of ongoing contamination.^59^ Farmers in Preak Russey spend a lot of time maintaining their water pumps which is where the soil contamination with As is the most extreme. Assessments of Zn deficiency in farmers in Preak Russey would help to manage their health risks and provide a baseline to evaluate the effectiveness of any improved management.

### Irrigation water

The FAO guidelines for As in irrigation water is 100 μg/L and the wells in Preak Russey have 10 times this level of As *(Table 3)* (953±349 μg/L).^4^ This FAO guideline is outdated (~1992) and does not reflect the greater need to protect children compared to the general public.^4^ In countries where rice consumption is high, guidelines for As in irrigation water are lower. In Japan, the guideline for As in irrigation water is 10 μg/L.^60^ In Taiwan and Korea, the guidelines for As in irrigation water are both 50 μg/L.^61,62^ These guidelines were developed with extensive risk analysis to protect crop productivity, ensure food quality and protect human health. The 10- to 100-fold exceedances of international irrigation guidelines and as much as 2-to 3-fold exceedances of EU children's guidelines for food should be enough justification for further remediation in Preak Russey.^4,60–62^

Most farmers want to avoid As, but lack better water for irrigation. There is a shortage of funding, but the problems related to As are closely linked to the management of climate change, food production, the economy and human health.^63^ Water storage is essential to managing increasingly variable fluctuations in rainfall that reflect climate change and better supplies of irrigation water are important to curb the use of groundwater for growing rice. Water storage and irrigation are expensive, and novel engineering and funding mechanisms related to climate change are required. A full review is beyond the scope of this paper.

There is no doubt that most of the bioaccumulated As comes from the irrigation wells.^8^ There is also little doubt that most of the As applied to the fields leaves by erosion, downward leaching or perhaps at times by volatilization of trimethyl arsine gas, but the pathways are inadequately understood.^64–66^ It is possible to optimize As removal in the water distribution ditches rather than the common irrigation approach of directly pumping groundwater into the paddy field where As detoxification reactions proceed less efficiently in the rice paddy fields.^8^

Two treatment (water distribution) ditches in Preak Russey removed 95% of the As in groundwater. The farmer who, by chance, discovered the treatment ditch approach at site Preak Russey-4 produced brown rice with 159 μg/kg inorganic As.^8^ The Zn content of Preak Russey-4 rice was the 3^rd^ highest observed in the present study *(Figure 4).* These ditches also removed 92–99% of the Fe prior to irrigation of the field.^1^ Enough Fe is added by the irrigation water (Preak Russey, 9565±6635 μg/L (n=20)) to turn parts of the paddy soil red or yellow. Added Fe is likely a contributing cause of Zn deficiency and Fe is toxic to rice, especially when the redox is low.^67–70^ The treatment ditch that we first observed in 2016 was still used at Preak Russey-4 in 2019. The ditch is effective, but uncertainties remain about the cost of repumping the water an extra time and there are concerns about the amount of land required for the ditch. The process could likely be optimized to reduce the amount of land and pumping requirements. There are costs associated with maintaining ditches. Ditches also provide significant water storage and are essential for periodic oxidation of the soils, mediated by field drainage. Storing water in upstream wetlands should be evaluated using methods that are coordinated with government fisheries agencies. Treatment of irrigation water or use of other sources of As-free water is required to reduce As bioaccumulation in children.

### Field drainage

Improved irrigation supplies are required before consideration of either field drainage or fertilizer enrichment. Long periods of flooding rice without drainage contributes to Zn deficiency.^1^ However, studies in the United States have found that application of Zn sulphate without first draining the field for oxidation was not effective.^71^ In the United States, fields were left drained for two weeks; likely it would be the same in Cambodia, but validation is needed. Without oxidation enabled by drainage, the precipitation of Zn sulphide would inactivate the Zn. Furthermore, in parts of both the United States and Australia, the usual treatment for rice straighthead disease is mid-season drainage of rice paddies.^72,73^ The most significant effect of redox on rice in the Preak Russey-1 and Preak Russey-5 sites appears to be the 3 to 4-fold higher levels of As(III), either as a proportion of inorganic or total As, respectively.^1^ It could indicate a lower redox in the Preak Russey-1 soils. This aspect should be better managed with clarification provided through mid-season drainage that include redox measurements to guide the drainage. Only one farmer said he understood the need for drainage of his field (Preak Russey-2). In 20 field trips, Preak Russey-2 and Preak Russey-9 were the only farms where we could see the paddy water had either seeped through the berm or had been drained. There are no studies with redox measurements to guide the drainage. Moreover, the only time in the 20 trips that we observed the rice fields as being dry was at harvest time. For most farms, paddy field drainage was not well managed. Farmers do not have adequate access to irrigation water and are unwilling to drain their fields and lose their water. Clearly, better irrigation is required before optimization of drainage with redox monitoring and analysis of the As and Zn content of the produced rice.

### Organic matter in soils

**Figure 6:**
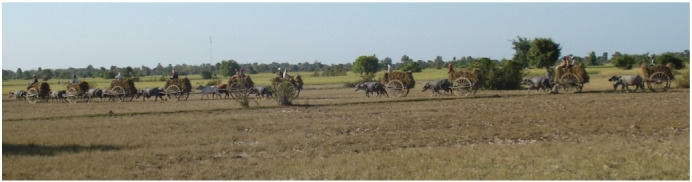
Rice harvest 2009, Bakan Cambodia

**Figure 7:**
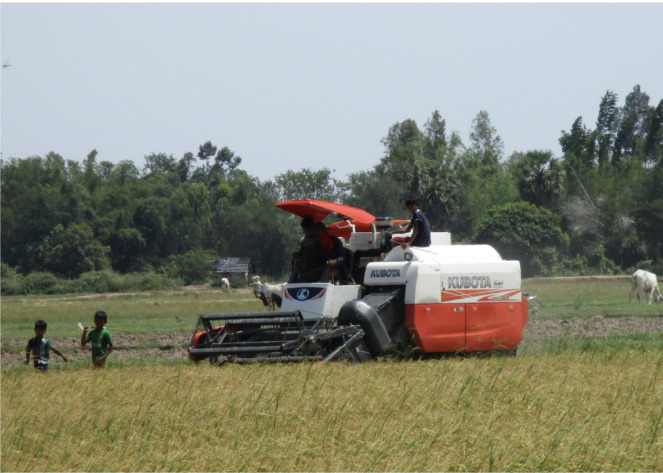
Rice harvest 2016, Preak Russey

The ongoing changes in rice cultivation associated with mechanization, irrigation, use of chemical fertilizers, and increased cropping frequency are commonly believed to result in lower levels of organic matter in soil, but there is a lack of historical data in Cambodia to validate this idea. Changes in land management using less livestock supports this hypothesis. Increasing the organic content of soils, as with manures, enhances the availability of Zn.^74–76^ The present study observed a significant increase in Zn content of rice from farms with cattle that use manure on their fields *(Figure 4).*^1^ However, the benefit of manure can be mitigated, as organic matter can enhance the availability of As.^77^ Decades ago, cattle and buffalo must have been a key component in maintaining Zn bioavailability without groundwater irrigation, and As would not have been as serious a problem as it is now. While the addition of manure illustrates that field management can affect Zn bioavailability, likely other variables such as irrigation and field drainage are more important. Today, many farmers lack the required manpower to maintain cattle. To illustrate these changes, a harvest scene from 2009 is presented in Figure *6*, showing a typical farm (Bakan, Cambodia) compared to the harvest in 2016 at Preak Russey *(Figure 7).* Figure *6* shows a team that harvested 1 ha of rice in one day; 14 adult Cambodians, and 18 buffalo or oxen pulling 10 carts. In Figure 7, one combine and a few adults harvested one ha in much less than a day.

New approaches are required to maintain the organic content of soils. The continued use of groundwater irrigation could compromise some options. Green manuring crops have been shown to increase the bioavailability of Zn to rice for soils in India.^78^ Green manuring is the growing of a cover crop to be later plowed into the soil to enhance the organic content. But if groundwater irrigation were to continue, green manuring might also enhance As bioavailability. In general, the old weathered As in soils is less bioavailable than the highly reactive As in groundwater. Any new approach for growing rice should be validated with As and Zn analysis of the rice. In Preak Russey, the former commune chief only used surface water for irrigation and his brown rice had the lowest level of inorganic As in the village (Preak Russey-15, 147 μg/kg As).^32^

### Study limitations

A greater number of samples from farms using surface water like Preak Russey-15 should be evaluated for a better understanding of the limitations of rice cultivation in this area. Evaluation of alternative rice cultivation methods (i.e. mid-season drainage) or potential substitution of rice with other crops such as wheat, corn or beans in the dry season would require substantial technical and social development. Ideally it would have been better to process all fields with more replicates, or to have representative integration of samples, but we had not realized the significant spatial effect that the wells produced and did not have the resources for extra analytical work, especially for speciation of As in rice. These limitations do not change any of our analyses, but future studies should be aware of the importance of the irrigation wells in further investigations.

Since the project began, the highways have improved and the daily commute to the site has been reduced by two hours, greatly enhancing the ease of sample collection. Likewise, since studies began on arsenic in Preak Russey, Cambodian universities have greatly improved, but laboratories still need updating, and there is a need for better electrical supplies. Collaboration on some analyses such as arsenic speciation is still needed from overseas laboratories. Similarly, Cambodian hospitals now perform some cancer analyses, but resources for medical analyses of farmers exposed to arsenic are lacking and some analyses are not available locally. Many safety issues associated with arsenic exposure, especially trimethylarsine, remain poorly understood.

### Possibility of improved rice cultivation via climate change initiatives

A Chinese review found that mid-season drainage of rice could reduce global warming potential (methane and nitrous oxide) of Chinese farmers by 47%.^79^ In 2019, the Japanese government sponsored an extension of the common mid-season water drainage in 20 000 ha of Japanese rice paddies. An additional week of drainage beyond the common two weeks decreased emissions of Japanese farmers' greenhouse gases (total of methane and nitrous oxide) by a further 30% over regular mid-season rice field drainage.^80^ This Japanese concept has also expanded to India.^80^ One study was critical of the Japanese project in that the main incentive, reducing climate change, has limited financial benefit to the farmer. The proposed sponsorship should reflect the risks and be more generous to encourage more farmers to cooperate.^81^ The risks include enhanced weed growth that may lead to reduced harvest, the potential need for more nitrogen fertilizer, and concerns about the availability of water to restore rice fields.^82^ Laboratory evaluations in a Danish growth chamber found that early season drainage of rice is also effective at reducing the release of greenhouse gases.^83^ In 2013, Murphy *et al.* stated that for Cambodian farmers to reduce the emission of greenhouse gases, they required the equivalent of carbon credits, ideally traded on world markets.^63^ The concepts of climate change mitigation are well understood. Improved management of rice irrigation would certainly improve Zn deficiency. The degree of enhancement of Zn bioavailability will depend on the type of irrigation, method of water drawdown, and other farm management measures such as fertilization, green manuring, type of rice grown, etc. Ideally, there would also be some financial incentive for farmers to optimize the Zn content of rice. The development of optimal management of Zn in Cambodian rice could take several years. However, there is no reason to delay attempts to secure carbon credits to improve irrigation, avoid using As-rich groundwater and enhance the Zn content of rice. It would be profoundly useful to step this up to an international project so that farmers in Cambodia could improve the quality of their rice crops, strengthen their health, and mitigate global climate change.

## Conclusions

Zinc deficiency and As bioaccumulation in rice are aggravated by irrigation with As-rich groundwater. Cow manure is able to increase the Zn content of rice, but other strategies such as improved irrigation, green manuring or perhaps direct augmentation of diet are required to increase Zn in the human diet. The curvilinear response of high DMA concentrations to high levels of total As *(Figure 3)* reflects greater toxicity from As and results in inadequate levels of Zn.

### Recommendations

The authors recommend alternative sources of surface water be provided to avoid using groundwater irrigation in Preak Russey, Cambodia. Treatment ditches should be optimized for As removal. Drainage of fields should be optimized using redox measurements, and analysis of Zn and As in rice grain should be conducted. The analysis of Zn deficiency in rice should be expanded to other areas of Cambodia.
